# Rethinking Research Upsurge of 6PPD-Quinone: Future Directions and Challenges for Hazard Evaluation and Pollution Control

**DOI:** 10.1021/envhealth.6c00169

**Published:** 2026-04-20

**Authors:** Yanhao Zhang, Shusheng Zhang, Zongwei Cai

**Affiliations:** † School of Ecology and Environment, 12636 Zhengzhou University, Zhengzhou 450001, China; ‡ College of Chemistry, Zhengzhou University, Zhengzhou 450001, China; § Eastern Institute of Technology, Ningbo 315100, China; ∥ State Key Laboratory of Environmental and Biological Analysis, 26679 Hong Kong Baptist University, Kowloon, Hong Kong SAR 999077, China

## 1 Introduction

At the end of 2020, an article published in *Science* reported a tire-rubber-derived toxic chemical, namely, *N*-(1,3-dimethylbutyl)-*N*′-phenyl-*p*-phenylenediamine-quinone (6PPD-quinone),^1^ triggering global attention and research upsurge. This compound has been identified as the culprit behind the acute mortality of adult coho salmon (*Oncorhynchus kisutch*) in U.S. Pacific Northwestern urban creeks following stormwater exposure, a phenomenon termed “urban runoff mortality syndrome”. Median lethal concentration (LC_50_) of 6PPD-quinone is only 95 ng L^–1^ for coho salmon.^2^ The low LC_50_ and coho salmon’s death indicate a considerably high possibility of environmental concentration meeting this threshold. Significant toxicity motivates massive investigations of 6PPD-quinone in ecosystems (e.g., air, soil, particles, snow) and food chains, not only in water.^3–5^ The latest evidence also demonstrates its occurrence in human cerebrospinal fluid^6^ and urine,^7^ which suggests 6PPD-quinone is transferring from the environment to the human body. These findings further prompt the expanding of 6PPD-quinone’s toxicological study, not only for coho salmon but also for other living organisms and experimental cell lines. Literature regarding the biotoxicity of 6PPD-quinone has had marked growth since 2021. Yet, a key question is, for other biological targets, whether they also have significant toxicities (e.g., acute death of coho salmon) after exposure to 6PPD-quinone under real environmental conditions. Another question is whether or not it is necessary to initiate the pollution treatment and control of 6PPD-quinone.

## 2 Necessity to Study Species-Specific Toxicity of 6PPD-Quinone

Indeed, 6PPD-quinone shows significant species-specific toxicity with environmental-related exposure dosages. While it can affect behaviors and organs of certain organisms, such as *Caenorhabditis elegans* (Nematoda), earthworms (Annelida) and mice/rats (Mammalia), the resulting toxicological outcomes (e.g., lifespan reduction, *in vivo* signaling pathway disorder and organ injury) are not acutely lethal, even at experimental dosages comparable to or higher than environmental levels. For representative Salmoniformes, including brook trout (*Salvelinus fontinalis*), rainbow trout (*Oncorhynchus mykiss*)^8^ and white-spotted char (*Salvelinus leucomaenis Pluvius*),^9^ 6PPD-quinone can induce their death but the exposure dosages and 24 h-LC_50_ are higher than 95 ng L^–1^. No mortality is observed for southern Asian dolly varden (*Salvelinus curilus*), landlocked masu salmon (*Oncorhynchus masou masou*)^9^ and Arctic char (*Salvelinus alpinus*).^8^ There is no doubt that, even within Salmoniformes, coho salmon is the most sensitive to 6PPD-quinone. Despite having massive toxicological data, no abnormal death or apparently untoward reactions induced by 6PPD-quinone have been found in real-world environments for any species other than coho salmon to date. The toxicity comparisons between Salmoniformes and other organisms, as well as among Salmoniformes themselves, consistently point out a potentially unique and different mechanism underlying the acute death of coho salmon induced by 6PPD-quinone. The disruption of vascular permeability^10^ and blood–brain/blood–gill barrier^11^ of coho salmon reported in previous work cannot fully account for the pronounced toxicological selectivity of 6PPD-quinone. It remains unclear whether this phenomenon stems from the ecological habits of coho salmon, molecular mechanism following exposure, or other contributing factors. In light of these uncertainties, we thus advocate for further exploring the “coho-salmon-selective” toxicological targets or mechanisms of 6PPD-quinone by integrated strategy of computational simulation (e.g., QSAR, network toxicology), chemical/systematic biology analysis (e.g., mass-spectrometry-based multiomics, affinity pull-down assays, bioinformatics) and biological validation (e.g., gene knockdown and overexpression) (Figure 1). Considering that selective toxicity may be influenced by multiple factors, it is recommended to use collected actual creek water for exposure experiments. The conditions of this creek water should be adjusted to simulate the aquatic physicochemical properties during large-scale coho salmon mortality events. For such complex biological experiments, machine learning and artificial intelligence can facilitate the extraction of critical information, and assist to find the potential association between toxicity and exposure parameters. Elucidating explicit selective targets/mechanisms can help us further predict affected organisms in theory under real environmental conditions, purposefully carrying out *in vivo* and *in vitro* hazard evaluation.

**Figure 1:**
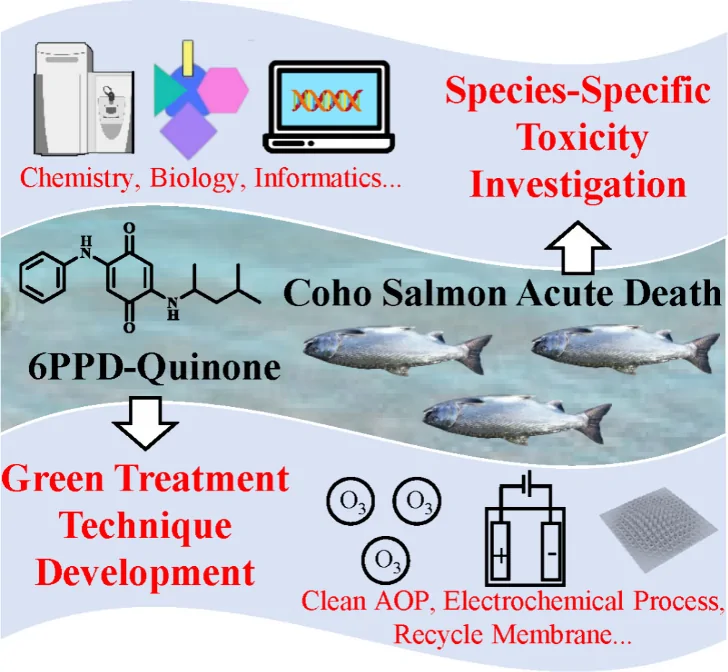
Various pathways for exploring “coho salmon-selective” toxicity and green treatment techniques of 6PPD-quinone.

## 3 Green Control and Treatment of Aquatic 6PPD-Quinone

The ubiquitous detection of 6PPD-quinone suggests its long-term accumulation in the ecological cycle. Considering 6PPD-quinone’s considerably toxicological impacts and the trophic position of coho salmon within the aquatic food web, we believe priority should be given to the treatment and control of aquatic 6PPD-quinone. It is a promising yet still problematic endeavor. Utilization of solar-light-activated sodium periodate (NaIO_4_, 20 mmol L^–1^) can degrade and detoxify environmental-level 6PPD-quinone,^12^ as shown in the first work focusing on treating this new pollutant by advanced oxidation process (AOP) in the laboratory. Although this AOP approach achieves highly efficient degradation (removal rate >99%) and detoxification, it introduces additional NaIO_4_, which has potential aquatic toxicity for golden pompano (*Trachinotus ovatus*).^13^ For practical application of the solar/IO_4_^–^ system in daily lives, a complicated aquatic environment raises concerns regarding unintended toxic effects on nontarget organisms. Treating 6PPD-quinone in an aquatic system must therefore be accompanied by efforts to prevent or mitigate secondary pollution. General catalytic degradation and adsorption removal approaches using functional materials (e.g., MOF, COF, polymer), AOP or microorganisms all can potentially bring additional burden into the aquatic system. Accordingly, we recommend development of green remediation strategies tailored to practical application scenarios to minimize secondary risks. Potential directions include the use of a relatively clean AOP (e.g., UV/O_3_) system and an electrochemical process for aquatic 6PPD-quinone treatment. Another viable pathway involves the deployment of recyclable devices, such as an advanced-material-based membrane, which should achieve effective degradation/adsorption removal and detoxification of 6PPD-quinone in a real aquatic environment; meanwhile, it is safe to other organisms and can be recovered to prevent extra pollution (Figure 1).

## 4 Conclusion and Outlook

While a large number of monitoring studies have demonstrated the extensive occurrence of 6PPD-quinone in the environment, there is no systematic guideline of its hazard evaluation and pollution control. This calls for expedited associated research. However, unlike certain notorious new pollutants, such as per- and polyfluoroalkyl substances (PFAS) with broadly known multispecies toxicities and adverse human health effects, 6PPD-quinone exhibits obvious species-specific toxicity. Its apparent damage is not currently found for the human body, which may lead to lower public awareness of its risks. Moreover, 6PPD, the generation precursor of 6PPD-quinone, is still universally employed as an antioxidant in the rubber industry. Washington State, USA, passed the bill 5931 in 2024, which supervises but does not prohibit 6PPD. In the same year, the U.S. Environmental Protection Agency launched an agency-wide action plan, encompassing detection method development, survey, risk assessment and municipal stormwater management for 6PPD and 6PPD-quinone. In 2026, a proposal focusing on controlling 6PPD-quinone was submitted to NPC and CPPCC sessions, which is the first action on this new pollutant in China. Although some major countries around the world intend to begin regulating 6PPD-quinone, it is a long-term process for government and society. The pollution of 6PPD-quinone will last with great possibility in the near future. Hence, despite multiple obstacles and challenges ahead, it is imperative to accelerate research into its species-selective toxicological mechanisms and to advance the development of green treatment technologies for aquatic environments. These efforts are essential for ultimately establishing regulatory and control frameworks capable of alleviating the ecological impacts of 6PPD-quinone.
